# Effects of Curcumin on Lipid Membranes: an EPR Spin-label Study

**DOI:** 10.1007/s12013-020-00906-5

**Published:** 2020-04-01

**Authors:** Mariusz Duda, Kaja Cygan, Anna Wisniewska-Becker

**Affiliations:** 1 Faculty of Biochemistry, Biophysics and Biotechnology, Jagiellonian University, Gronostajowa 7, 30-387 Kraków, Poland; 2 Malopolska Centre of Biotechnology, Jagiellonian University, Kraków, Poland, Gronostajowa 7A, 30-387 Kraków, Poland

## Abstract

Curcumin is a yellow–orange dye widely used as a spice, food coloring and food preservative. It also exhibits a broad range of therapeutic effects against different disorders such as cancer, diabetes, or neurodegenerative diseases. As a compound insoluble in water curcumin accumulates in cell membranes and due to this location it may indirectly lead to the observed effects by structurally altering the membrane environment. To exert strong structural effects on membrane curcumin needs to adopt a transbilayer orientation. However, there is no agreement in literature as to curcumin’s orientation and its structural effects on membranes. Here, we investigated the effects of curcumin on lipid order, lipid phase transition, and local polarity in a model liposome membranes made of DMPC or DSPC using electron paramagnetic resonance (EPR) spin labeling technique. Curcumin affected lipid order at different depths within the membrane: it slightly increased the phospholipid polar headgroup mobility as monitored by spectral parameters of T-PC, while along the acyl chain the ordering effect was observed in terms of order parameter S. Also, rotational correlation times τ_2B_ and τ_2C_ of 16-PC in the membrane center were increased by curcumin. Polarity measurements performed in frozen suspensions of liposomes revealed enhancement of water penetration by curcumin in the membrane center (16-PC) and in the polar headgroup region (T-PC) while the intermediate positions along the acyl chain (5-PC and 10-PC) were not significantly affected. Curcumin at a lower concentration (5 mol%) shifted the temperature of the DMPC main phase transition to lower values and increased the transition width, and at a higher concentration (10 mol%) abolished the transition completely. The observed effects suggest that curcumin adopts a transbilayer orientation within the membrane and most probably form oligomers of two molecules, each of them spanning the opposite bilayer leaflets. The effects are also discussed in terms of curcumin’s protective activity and compared with those imposed on membranes by other natural dyes known for their protective role, namely polar carotenoids, lutein and zeaxanthin.

## Introduction

**Fig. 1:**
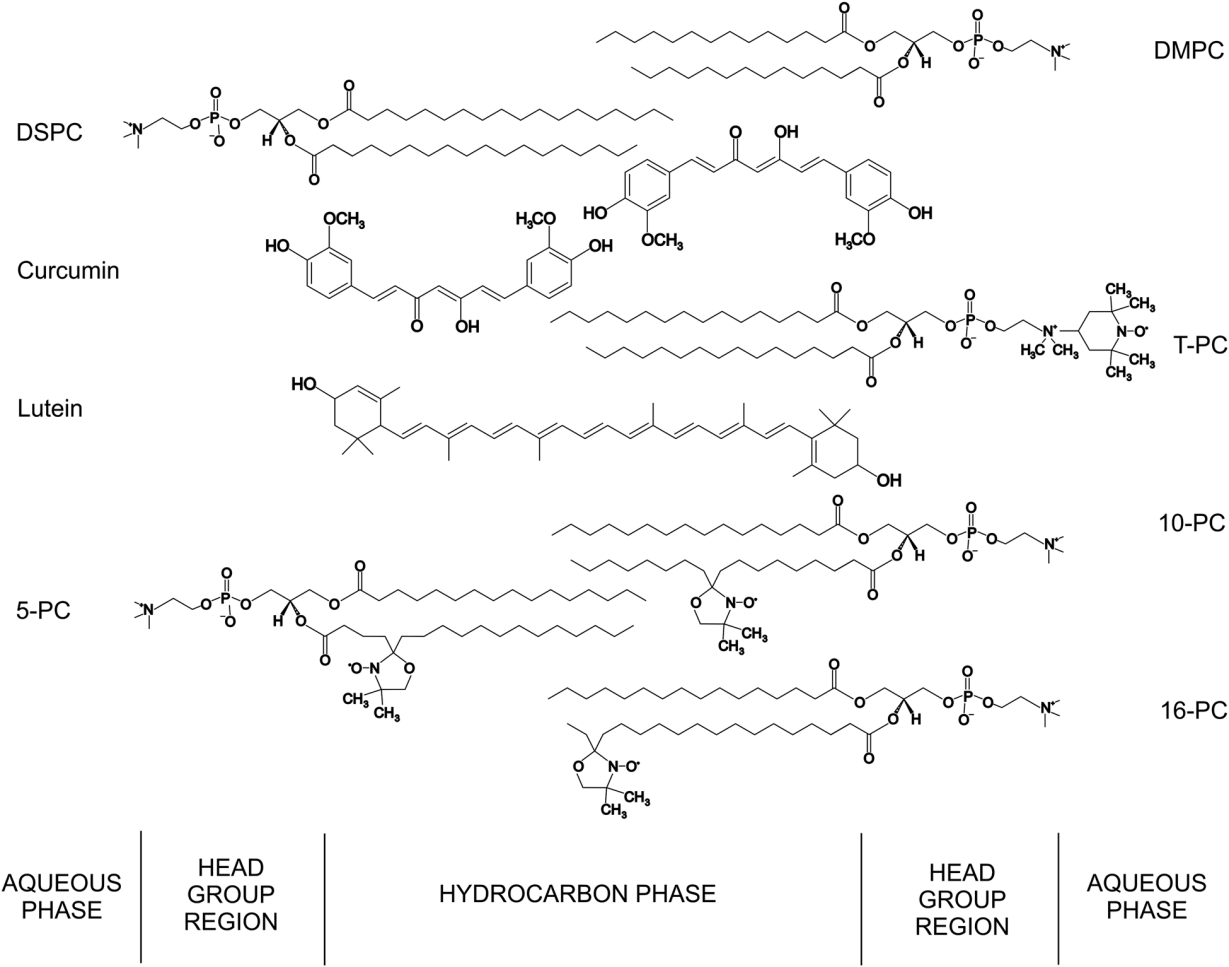
Cross-sectional drawing of a lipid bilayer formed by DMPC or DSPC including 5-, 10-, 16-PC and T-PC-spin labels. Approximate locations of lutein and curcumin across the lipid bilayer are also illustrated

Curcumin is a yellow–orange dye derived from the rhizome of Curcuma longa (check Fig. 1 for its chemical structure). The powdered root of this plant, turmeric, is widely used as a spice, food coloring, dye, and food preservative [1]. Curcumin has been shown to exhibit a broad range of therapeutic effects against different disorders, such as cancer, diabetes, or, more recently, neurodegenerative diseases [2–5]. It is not clear how curcumin exerts these many different effects. However, as a compound insoluble in water curcumin has affinity to lipids, and therefore associates with or accumulates in cell membranes [6–8]. Based on the accumulation of curcumin in membranes on one hand, and its wide spectrum of pharmacological properties on the other, it was suggested that curcumin may indirectly lead to the observed effects by altering the membrane environment. For example, curcumin may affect membrane proteins’ functions by modulating the host lipid bilayer properties [6]. By modulating those properties, curcumin may also make membrane less prone to oxidative stress. Because of that, curcumin’s way of action was often compared with that of cholesterol [9–12]. Similar to cholesterol, curcumin was proposed to be anchored to the lipid bilayer near the phosphate group and to stiffen membranes by inducing segmental ordering of lipids [9].

However, bearing in mind its origin and character, it seems more reasonable to compare curcumin with carotenoids, especially with those having hydroxyl polar groups at the ends of their molecule, such as xanthophylls lutein or zeaxanthin (Fig. 1). Curcumin and carotenoids are both natural lipophilic dyes known for their protective activity, both are recommended in diet, as supplements or from natural sources, and both affect membrane structure [8, 9, 13–15]. Although it is generally accepted that unique photoprotective properties of carotenoids stem mainly from their ability to effectively quench singlet oxygen, affecting membrane structural properties as another mechanism of their protective action was also suggested [16–19].

While analyzing the structural mechanism of membrane protection by carotenoids or curcumin, one has to consider the orientation of these compounds within the membrane. One molecule of lutein or zeaxanthin is long enough to span the whole lipid bilayer—two hydroxyl groups at the ends of a conjugated hydrocarbon “bar” of a carotenoid molecule are separated by the distance of about 30 Å (Fig. 1) [13, 20]. A xanthophyll molecule orients within the membrane mostly perpendicular to the surface being anchored in polar headgroup regions on both sides of the membrane by its hydroxyl groups, while location of apolar β-carotene is not clearly determined [15, 17, 21, 22]. This is the reason why xanthophylls are more effective than β-carotene in affecting membrane properties and protecting membrane lipids against peroxidation [16, 21, 23, 24]. Curcumin orientation in the membrane is under debate. Some results suggest that curcumin lies flat on the lipid headgroups, where it forms hydrogen bonds with the lipid molecules [25], whereas some other show that curcumin can penetrate deeply into the membrane and intercalate with the lipid tails [6, 9]. According to one of the recent papers, both locations are possible depending on membrane hydration [11]. Simulation studies showed that curcumin distribution in the membrane depends on the lipid type [26]. A combination of solid-state NMR and differential scanning calorimetry experiments showed that curcumin adopts a transbilayer orientation due to forming a hydrogen bond with the phosphate group of lipids [9]. The length of a curcumin molecule is estimated to be about 16 Å [27, 28]. Compared with a carotenoid molecule, it is about half shorter, therefore if embedded in the membrane, it can span only one leaflet of the lipid bilayer (Fig. 1). Depending on curcumin location in the membrane, different models of its protective activity are proposed: as a physical barrier, a so-called carpet, preventing peptide or oxidants penetration if associated with the membrane at the surface or by membrane stiffening and increasing the energy barrier to ion or peptide embedding if positioned perpendicular to the membrane surface and penetrating between the alkyl chains of lipids [11].

Here, we investigated the effects of curcumin on lipid order, lipid phase transition and local polarity in model liposome membranes made of 1,2-dimyristoyl-sn-glycero-3-phosphocholine (DMPC) or 1,2-distearoyl-sn-glycero-3-phosphocholine (DSPC) using electron paramagnetic resonance (EPR) spin labeling technique. Judging from the effects exerted by curcumin at different depths within the membrane, as well as in the membrane polar headgroup region, curcumin adopts a transbilayer orientation. Observed effects are also discussed in terms of curcumin’s protective activity.

## Materials and Methods

### Liposome Preparation

DMPC, DSPC, curcumin (1,7-bis(4-hydroxy-3-methoxyphenyl)1,6-heptadiene-3,5-dione) and 16-doxyl stearic acid (16-SASL) were purchased from Sigma-Aldrich, Poland, and spin labels: 1-palmitoyl-2-stearoyl-(n-doxyl)-sn-glycero-3-phosphocholine (n-PC, where *n* = 5, 10, or 16) and TEMPO-PC (1,2-dipalmitoyl-sn-glycero-3-phospho(tempo)choline) from Avanti Polar Lipids, Alabaster, USA.

The membranes used in this work were multilamellar liposomes consisting of DMPC or DSPC and respective amount of curcumin. The liposomes were prepared by the following method [29]. Briefly, chloroform solutions of lipids (containing 2.5 μmol of total lipid), curcumin (5 or 10 mol%, if applicable), and spin labels (1 mol%) were mixed, chloroform was evaporated with a stream of nitrogen, and the lipid film on the bottom of the test tube was thoroughly dried under reduced pressure (about 0.1 mm Hg) for 12 h. A phosphate buffered saline pH 7.4 (usually 0.5 mL) was added to the dried film at the temperature well above the lipid phase transition temperature (T_M_) and vortexed vigorously. Then, the multilamellar liposome suspension underwent five freeze–thaw cycles, after which it was centrifuged at 14,000 × *g* for 15 min at 4 °C, and the resulting pellet was used for EPR measurements.

### EPR Measurements

T-PC, 5-PC, 10-PC, and 16-PC are phospholipid spin labels that have a nitroxide free radical moiety responsible for the EPR signal attached to the polar headgroup, or to the 5th, 10th, or 16th carbon atom in the acyl chain, respectively. Therefore, information is obtained from different regions of the membrane: the water–membrane interface, the region close to the polar headgroups and the membrane center (Fig. 1). The EPR measurements were performed with Bruker EMX spectrometer equipped with a temperature control unit (EMX ER 4141 VT). The suspension of multilamellar liposomes containing 1 mol% of a spin label was placed in a gas permeable capillary (i.d. 0.7 mm) made of TPX and located inside the EPR dewar insert in the resonant cavity of the spectrometer. The sample was thoroughly deoxygenated with nitrogen gas (about 15 min), which was also used for temperature control.

**Fig. 2:**
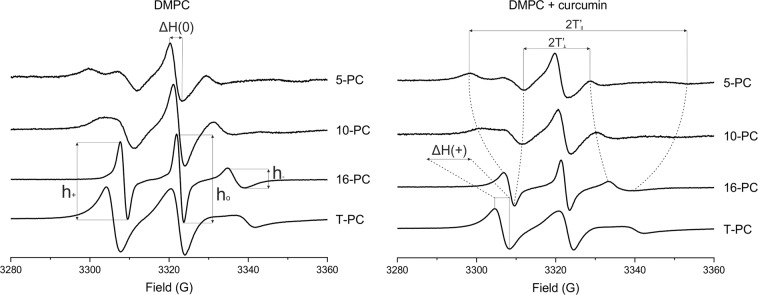
EPR spectra of 5-,10-,16-PC, and T-PC spin labels in DMPC liposome membranes in the absence (left panel) and presence (right panel) of 10 mol% curcumin obtained at 25 °C. Measured values are indicated: the peak-to-peak distances (2 T_II_ and 2T_┴_) used for calculating the order parameter S, the width of the central line (ΔH(0)) and the amplitudes of all three lines of 16-PC spectra (h_0_, h_−_, h_+_) used for calculating the rotational correlation times and the width of the low-field line (ΔH(+)) and the amplitudes of the central (h_0_) and high-field (h_−_) lines of T-PC spectra

For measurements of lipid order, the EPR spectra were recorded at 25 and at 37 °C for DMPC, and at 60 °C for DSPC. For DMPC phase transition measurements, the temperature was regulated with the accuracy of ±0.1 °C (between 22 and 24 °C). The measurements were done starting from higher temperatures. For polarity measurements, samples were frozen to 120 K (−153 °C) using liquid nitrogen vapor. Exemplary EPR spectra of the spin labels used in the study are shown in Fig. 2 together with the measured parameters.

## Results and Discussion

### Effect of Curcumin on Lipid Mobility in the Polar Headgroup Region

**Fig. 3:**
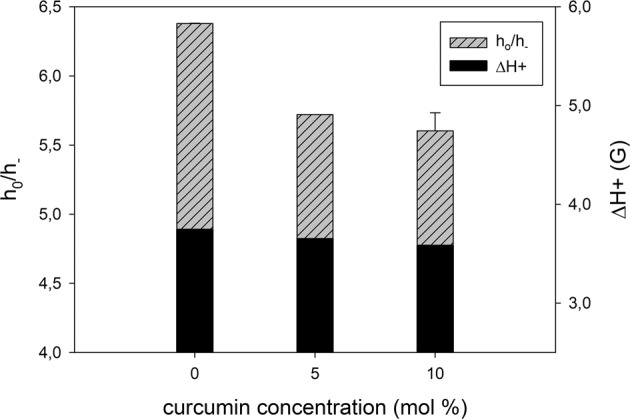
EPR spectral parameters of T-PC spin probe measured for DMPC membrane in the presence and absence of curcumin at 25 °C. h_0_/h_−_ is a ratio of the height of central and high-field peaks and ΔH+ is a peak-to-peak width of the low-field line

To investigate the polar headgroup region of DMPC and DSPC membranes T-PC spin probe was employed. The parameters which were obtained directly from the EPR spectra of T-PC: peak-to-peak width of the low-field line (ΔH+) and the ratio of the height of central and high-field peaks (h_0_/h_−_) (Fig. 2) give information about the headgroup motional freedom [13]. The increase in the motional freedom results in the decrease of these parameters [13, 30]. Both spectral parameters obtained at 25 °C have smaller values in the DMPC membranes containing curcumin than in the pure membranes (Fig. 3). Especially, a significant decrease of the (h_0_/h_−_) parameter shows that the anisotropy of the motion of the nitroxide moiety of T-PC is decreased in the presence of curcumin which can be interpreted as the increase in the mobility of the polar headgroups. The results suggest that in the presence of curcumin the polar headgroups of lipid molecules are more separated from each other and have more motional freedom. This effect is similar to that of cholesterol and zeaxanthin [13, 31, 32]. In DSPC membranes the effect of curcumin on polar headgroup mobility is negligible (data not shown). The lack of the effect of curcumin in this membrane can be explained by the necessity of performing the measurements at a high temperature (60 °C) because of a high T_M_ of DSPC (55° C) [33]. As shown previously, the effects of different compounds on lipid headgroups motion decrease with rising of temperature [13, 15, 30, 34]. Especially, the effect of zeaxanthin disappeared already at 45 °C [13].

Barry et al. [9] observed a conformational change in the phosphocholine headgroup of DMPC membrane in the presence of curcumin, which they ascribed to the forming of a hydrogen bond between the curcumin hydroxyl group and either the phosphate group or the nearby carbonyls of DMPC. Analogous hydrogen interaction was shown for polar carotenoids and suggested to be responsible for their transbilayer orientation [22, 35]. Grudzinski et al. [22] showed that the vertical arrangement of xanthophylls is stabilized by the formation of hydrogen bonds between the terminal hydroxyl groups located in the C3 and C3′ positions with lipid polar headgroups which constitute the two opposite polar membrane regions. Such an anchoring effect of hydroxyl groups seems to be present also in case of curcumin. One has to remember however that a molecule of curcumin spans only one leaflet of the bilayer, therefore the anchoring of curcumin at polar headgroup region does not necessarily imply its vertical orientation within the membrane. To check this, the effects of curcumin on the lipid acyl chains at different depths within the membrane had to be investigated.

### Effect of Curcumin on Lipid Acyl Chain Order and Mobility

**Fig. 4:**
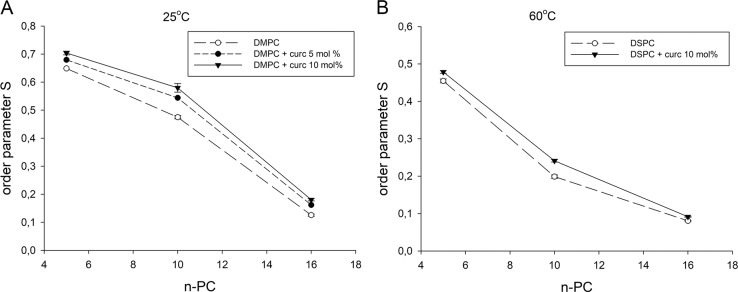
Order parameter S of 5-, 10- and 16-PC in DMPC (a) and DSPC (b) membranes in the presence and absence of curcumin

To study the effect of curcumin on the order of lipid acyl chains in DMPC and DSPC membranes, the order parameter S was calculated based on spectral parameters 2T_II_ and 2T_┴_ of 5-, 10- and 16-PC spin labels (Fig. 2) according to Marsh [36]. In the case of n-PC, S reflects the segmental order parameter of the hydrocarbon chain segment to which the nitroxide fragment is attached. As shown in Fig. 4, curcumin increases the order of acyl chains in every position within the DMPC (Fig. 4a) and DSPC (Fig. 4b) membranes. The effect is especially pronounced in the case of DMPC and in the membrane center where S parameter of 10-PC and 16-PC is increased in the presence of 10 mol% of curcumin by about 25% and 40%, respectively. In DSPC membrane the ordering effect of curcumin is less pronounced, but also the strongest in the membrane center (about 20% for 10-PC). Like in the case of lipid headgroup mobility, the lower effect of curcumin on S parameter in DSPC membrane can be explained by a high temperature of the measurement (60 °C).

**Fig. 5:**
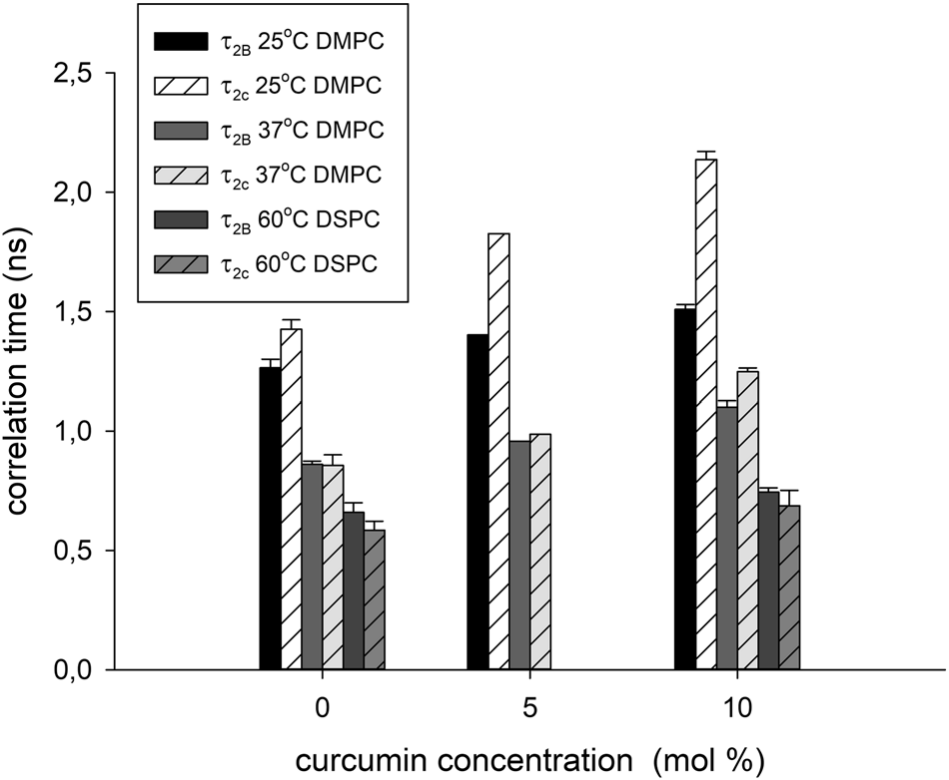
Rotational correlation times τ_2B_ and τ_2C_ of 16-PC in DMPC (25 and 37 °C) and DSPC (60 °C) membranes in the presence and absence of curcumin

A relatively fast motion of 16-PC spin label yields more isotropic spectra and allows calculation of the rotational correlation times τ_2B_ and τ_2C_ using the formulas provided by Berliner [37]. Curcumin clearly increases the rotational correlation times of 16-PC in both investigated membranes (Fig. 5). For example, at 25 °C τ_2B_ increases from 1.26 ns in the pure DMPC membrane to 1.4 ns and to 1.51 ns in the presence of 5 mol% and 10 mol% of curcumin, respectively. Changes in τ_2C_ are even more significant—from 1.43 ns in the pure DMPC to 1.83 ns and to 2.14 ns in the presence of 5 mol% and 10 mol% of curcumin, respectively. In addition, membranes containing curcumin exhibit a bigger difference between τ_2B_ and τ_2C_, which means that in the membrane center the motion of lipid acyl chains in the presence of curcumin is not only slower, but also more anisotropic than in the membranes without curcumin. The effects of curcumin on correlation times at higher temperatures (37 °C for DMPC and 60 °C for DSPC) are weaker, but not negligible.

Both approaches (S parameter and rotational correlation times) show rigidifying effect of curcumin on the DMPC and DSPC membranes. There is no agreement in literature on the curcumin’s effect on the order of membrane lipids as well as on its location in the membrane. One of the reasons might be a variety of membrane systems used. For example, Starok et al. [38] showed that curcumin insertion into the lipid bilayer of model membranes (EggPC/NBD-DPPE bilayer supported on a glass surface (SLB) as well as small unilamellar liposomes (SUV) composed of DPPC) leads to its rigidification. Barry et al. [9] suggested that curcumin induces segmental ordering in the membrane of DMPC bicelles, which resembles the effect of cholesterol. The results of ^2^H and ^14^N NMR measurements obtained by Kotenkov et al. [12] showed that curcumin is not embedded deep in the lipid bilayer of multilamellar liposomes and interacts mainly with the lipid headgroups. In the carpet model, according to which curcumin lies flat on the lipid headgroups it dehydrates lipid bilayers and decreases fluidity [11]. In the DMPC/cholesterol mixture addition of curcumin amplifies the effect of cholesterol on the ordering of lipid acyl chains [12]. Fluorescence quenching measurements showed that curcumin locates preferentially in the hydrophobic acyl chain region, close to the glycerol group of lipid molecules. Condensing effect of curcumin on EYPC monolayer without cholesterol was observed, whereas in EYPC monolayer containing cholesterol the effect was the opposite [8]. Different effects of curcumin on lipid order was observed also in the membranes without cholesterol. Alsop et al. [11] showed using X-ray diffraction technique on highly oriented multilamellar membrane stacks of DMPC that when inserted into the DMPC membrane, curcumin leads to a fluidification of the membrane and an increase in tail fluctuations, contrary to cholesterol’s condensing effect. Ingolfsson et al. [6] also showed that curcumin decreases bilayer stiffness, allowing easier forming of gramicidin A channels in “black” DOPC membranes. Also Hung et al. [7] suggested that curcumin can cause a nonlinear membrane thinning effect and affect gramicidin channels in multiple DOPC bilayers on quartz support.

Our results support the conclusion of Barry et al. [9] that curcumin has a strong effect on the dynamics of the lipid bilayer, altering the acyl chain packing and imparting order to most of the lipid molecule. However, contrary to their work, we observed the strongest ordering effect in the membrane center. This leads to the conclusion that curcumin is oriented perpendicular to the membrane surface, forming kind of oligomers consisting of two molecules each spanning an opposite bilayer leaflet (Fig. 1). The terminal rings of two curcumin molecules, each penetrating the opposite leaflets, most probably form hydrogen bonds with each other (between the hydroxyl groups) which stabilize the oligomers and a transbilayer orientation of curcumin. Although not observing the ordering effect of curcumin in the membrane center, Barry et al. [9] suggested that in the transbilayer orientation curcumin has unsatisfied hydrogen bonds that can be satisfied by the formation of oligomeric curcumin complexes. In addition, one has to consider here the difference in the thickness between the membranes of DMPC and DSPC and possible mismatch between the thickness of the membranes and the length of a curcumin molecule. This length (16 Å) is slightly less than one-half of the phosphate-to-phosphate length of a DMPC bilayer (34 Å), but much less than the same length of a DSPC bilayer (40.5 Å) [33]. Therefore, in the case of DSPC either curcumin sinks deeper in the bilayer preserving the hydrogen interaction between two curcumin molecules and stabilizing the transbilayer orientation, or stays anchored in the same position in the polar headgroup region but the hydrogen bonding in the center of the membrane breaks and the orientation is no more clearly determined. If the former takes place, we should observe strong effects of curcumin in the membrane center also in DSPC, whereas if the latter takes place curcumin should affect T-PC parameters the same way in both membranes, but in the membrane center the effect should be smaller in case of DSPC. Based on the results presented above it is difficult to conclude due to differences in temperature in which measurements in DMPC and DSPC were performed. However, polarity measurements in frozen suspensions of liposomes can shed additional light on the problem.

### Effect of Curcumin on Polarity Profiles

**Fig. 6:**
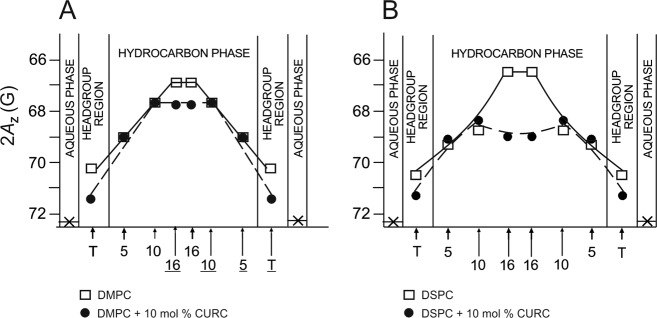
Polarity profiles across DMPC (a) and DSPC (b) membranes in the absence and presence of 10 mol% of curcumin. The spectra were acquired at −153 °C. Upward changes of 2Az indicate a decrease in polarity. Approximate locations of the nitroxide moieties of spin labels are indicated by arrows

2A_z_ parameter (z-component of the hyperfine interaction tensor) obtained from frozen suspensions of liposomes gives information about the polarity of the local environment in which a nitroxide free radical moiety of a spin label is placed [39]. Polar surroundings increases the unpaired electron density over the nitrogen atom and as a result the 2A_z_ splitting also increases [40]. Using the calibration plot of 2Az against the dielectric constant ε of a solvent, the local polarity in the membrane can be estimated (Fig. 2 in [39]). Polarity profiles of DMPC and DSPC membranes presented in Fig. 6 show that curcumin clearly increases water penetration in two membrane regions—deeply in the center and in the polar headgroup region. The effect is especially pronounced in the center of the DSPC membrane where the value of 2A_z_ parameter of 16-PC increases from 66.4G (comparable with the polarity of 1-decanol of *ε* = ~8) in the pure membrane to 69G (comparable with the polarity of methanol of *ε* = ~35) in the membrane containing 10 mol% of curcumin (Fig. 6b). In the presence of curcumin the profile across the hydrocarbon part of the membrane becomes flat showing no significant differences in polarity. In the polar headgroup region curcumin also increases polarity. In the DMPC membrane the value of 2A_z_ parameter of T-PC increases from 70.2 G (polarity level of formic acid of *ε* = ~60) in the pure DMPC membrane to about 71.3 G (polarity level of cyanoacetylene of *ε* = ~70) in the membrane containing 10 mol% of curcumin. In the DSPC membrane the changes are similar. This increase in water penetration into the polar headgroup region suggests that there is more free space between the polar headgroups. This result is in accordance with the increase in the polar headgroups motion observed for DMPC (Fig. 3). Interestingly, practically no effect of curcumin on both membranes polarity is observed for 5-PC and 10-PC. Bearing in mind the structure of a curcumin molecule, the polarity profiles confirm its transbilayer orientation. Assuming this orientation, the effect of increased water penetration is observed in the places where the terminal rings containing polar hydroxyl and methoxyl groups of curcumin are located. Similar effects of increased polarity were observed for membranes containing cholesterol where water penetration was increased up to 7–9th carbon atom, i.e., till the depth to which the cholesterol’s ring structure extends [39], and for membranes containing polar carotenoids, where water penetration was increased around the carotenoids’ terminal rings located in the polar headgroup regions of the opposite membrane sides [15, 29]. A comparison of the curcumin’s effects on the DMPC and DSPC membranes allows to conclude that the membrane thickness neither affects curcumin’s orientation within the membrane nor its tendency to form oligomers. Especially, the strong effect of curcumin on polarity in the center of the DSPC membrane suggests that the hydrogen bonding between two curcumin molecules spanning the opposite leaflets of the membrane is strong enough to stabilize the oligomer even if the membrane width and curcumin dimer length do not fully match.

### Effect of Curcumin on the Main Phase Transition of DMPC

**Fig. 7:**
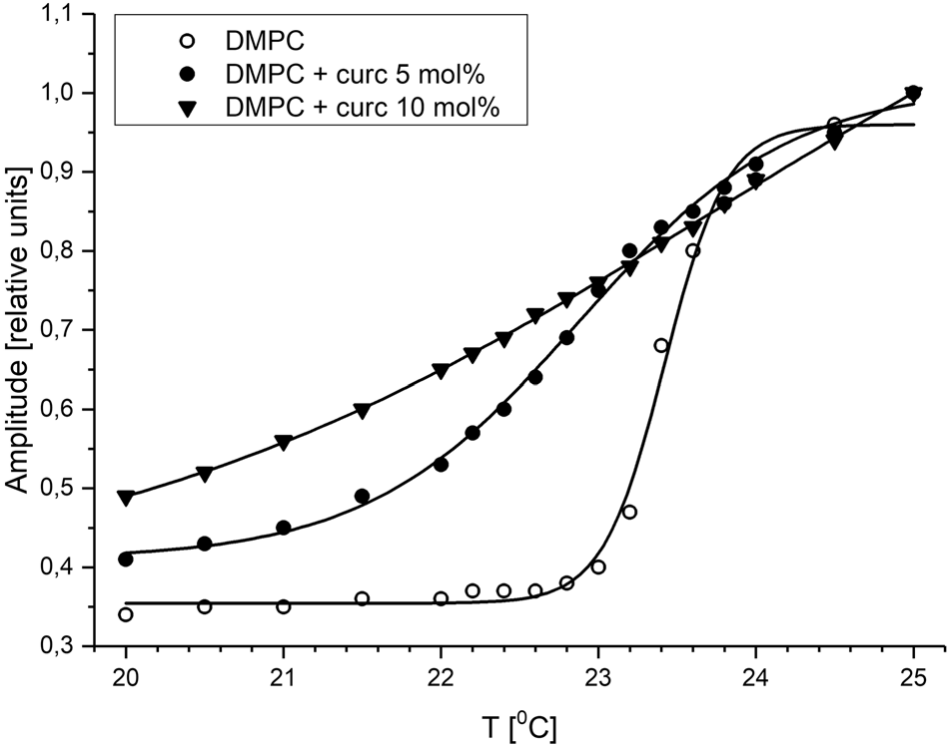
Determination of the DMPC phase transition temperature (T_M_) in the absence and presence of 5 and 10 mol% of curcumin using 16-SASL. Changes of the normalized EPR signal amplitude with temperature are depicted

The main phase transition temperature (T_M_) of DMPC obtained from measurements of the EPR signal amplitude of 16-SASL (Fig. 7) was 23.4 °C (Table 1), which is in agreement with literature [33, 41]. In the case of the pure DMPC membrane, the phase transition is narrow and cooperative (Table 1). Addition of 5 mol% of curcumin shifts the T_M_ to lower temperatures (22.9 °C), but more striking is its effect on the phase transition width, which increases significantly, about 3.5 times (from 0.2 to 0.7 °C). Curcumin in the concentration of 10 mol% abolishes completely the phase transition of DMPC and neither the T_M_ nor the dT_1/2_ were possible to determine. Again, the effect of curcumin on lipid phase transition is similar to the effect of polar carotenoids, which shift the T_M_ to lower temperatures and broaden the phase transition [15, 42]. Interestingly, β-carotene, which does not have a preferable orientation within the membrane has a minimal effect on lipid phase transition [15]. The effect of curcumin on lipid phase transition gives therefore an additional argument in favor of its transbilayer orientation. Barry et al. [9] drew a similar conclusion based on the comparison of curcumin with cholesterol. They stated that the effect of curcumin on the phase transition in DMPC was similar to that of cholesterol, which is known to adopt a transbilayer orientation.

**Table 1:** The main phase transition temperatures (T_M_) and the transition widths (dT_1/2_) of DMPC in the absence and presence of 5 and 10 mol% of curcumin

	T_M_ (°C)	dT_1/2_ (°C)
DMPC	23.4	0.2
DMPC +5 mol% curcumin	22.9	0.7
DMPC +10 mol% curcumin	n.a.	n.a.

## Conclusions

The results presented in this work showing strong effects of curcumin on membrane lipid order, polarity and phase transition suggest that curcumin adopts a perpendicular orientation within the membrane. Moreover, this orientation seems to be independent of the membrane thickness. This in turn suggests that hydrogen bonding presumably formed between terminal rings of two curcumin molecules spanning opposite leaflets of the lipid bilayer is strong enough to stabilize curcumin oligomers even when the length of curcumin dimer and thickness of the membrane do not match. The effects of curcumin on membrane properties are in many aspects similar to the effects of polar carotenoids, such as lutein or zeaxanthin. Curcumin and carotenoids both increase acyl chain order, separate polar headgroups and broaden and finally abolish the phase transition of membrane lipids. However, there is one significant difference between the effects exerted by these compounds. Polar carotenoids increase water penetration only in the headgroup region, and only in thinner membranes, such as DMPC. Within the membrane carotenoids increase the hydrophobicity barrier significantly. Curcumin increases water penetration not only in the headgroup region but also in the center of the membrane. This may have important consequences related to its protective properties. On one hand, increased lipid order should make membranes more resistant to penetration by different compounds, such as oxidants or peptides, on the other hand, increased water penetration may mean more water soluble free radicals or transition metal ions which can initiate or re-initiate lipid peroxidation. Carotenoids protect membranes structurally, increasing their rigidity but also forming a hydrophobic barrier to polar free radicals, redox-active metal ions or other oxidants, whereas curcumin provides no barrier of this type. Moreover, increased penetration of water and water soluble compounds in the presence of curcumin may make membrane not less but more susceptible to oxidative stress.
